# Supplementary material for: Zoonotic Malaria Risk in Serra Do Mar, Atlantic Forest, Brazil
Source: Microorganisms. 2023 Sep 30;11(10):2465. doi: 10.3390/microorganisms11102465 (PMC10609463; doi:10.3390/microorganisms11102465)
Supplement: Supplementary file 1 [file microorganisms-11-02465-s001.zip › microorganisms-2592554-supplementary.pdf]

Supplementary Material for the article Zoonotic Malaria Risk in Serra do Mar, Atlantic Forest, Brazil

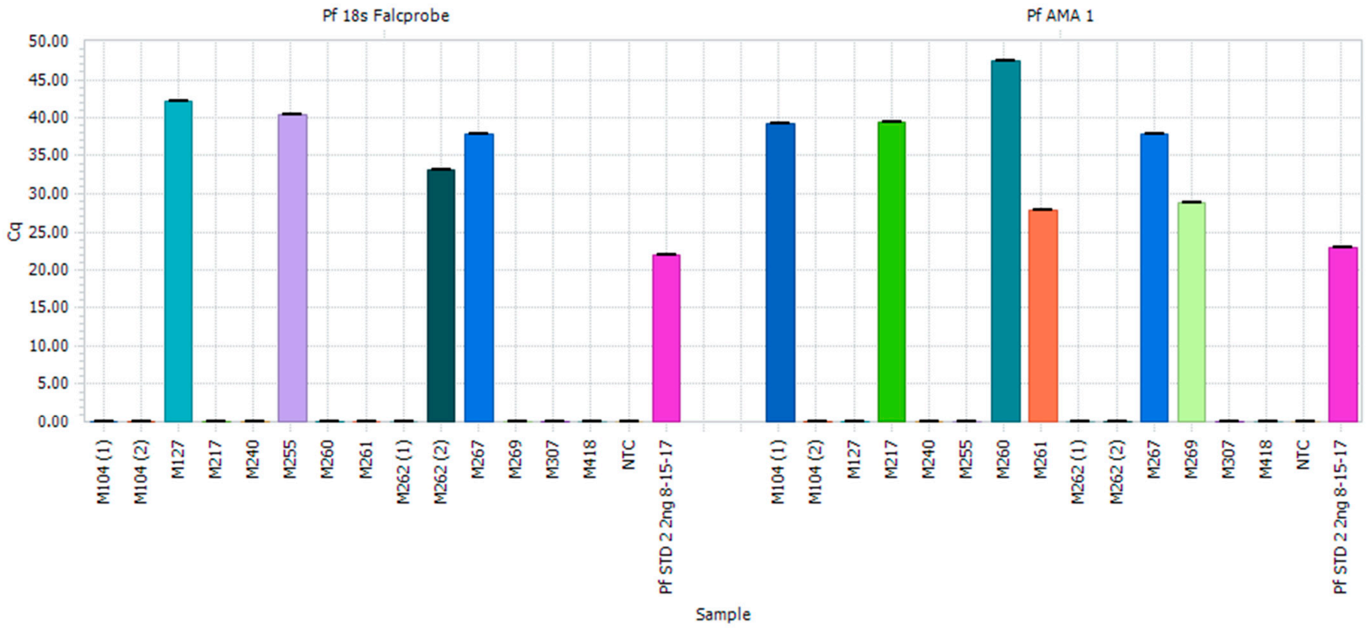

Figure S1. Outcomes from laboratory testing of samples of *Ke. cruzii* for *P. falciparum* at University of Florida, Gainesville FL, USA, December 2017. These samples went all positive in the work by Laporta et al. 2015 [1]. Sample n. *M267* went positive in both assays and its *cytb* sequence has been shown into a phylogenetic analysis [2] and is deposited in GenBank under accession number MT779800 and URL = https://www.ncbi.nlm.nih.gov/nuccore/MT779800.

Table S1. Specimens of *Ke. cruzii* captured in January 2019 in the Legado das Águas Forest Reserve and tested at the University of Florida, March 2019.

Species	Site	Longitude	Latitude	Collected/Tested[1]	Positive
Ke. cruzii	Cambuci Suspended Trail	-47.35294	-24.0317	365	0
Ke. cruzii	Alecrim Waterfall	-47.50002	-24.00602	1,161	0

[1]: testing involved *18S* qPCR for *P. vivax*, *P. malariae*, and *P. falciparum*.
